# Immune Dysfunction in Children with CHARGE Syndrome: A Cross-Sectional Study

**DOI:** 10.1371/journal.pone.0142350

**Published:** 2015-11-06

**Authors:** Monica T. Y. Wong, Annechien J. A. Lambeck, Mirjam van der Burg, Sacha la Bastide-van Gemert, Lianne A. Hogendorf, Conny M. A. van Ravenswaaij-Arts, Elisabeth H. Schölvinck

**Affiliations:** 1 University of Groningen, University Medical Centre Groningen, Department of Genetics, Groningen, The Netherlands; 2 University of Groningen, University Medical Centre Groningen, Department of Laboratory Medicine, Medical Immunology, Groningen, The Netherlands; 3 Erasmus MC, University Medical Centre Rotterdam, Department of Immunology, Rotterdam, The Netherlands; 4 University of Groningen, University Medical Centre Groningen, Department of Epidemiology, Groningen, The Netherlands; 5 University of Groningen, University Medical Centre Groningen, Beatrix Children’s Hospital, Department of Paediatrics, Infectious Diseases and Immunology section, Groningen, The Netherlands; George Washington University, UNITED STATES

## Abstract

CHARGE syndrome is a variable, multiple congenital malformation syndrome. Patients with CHARGE syndrome have frequent infections that are presumed to be due to anatomical anomalies of the craniofacial region and upper airway, and cranial nerve problems resulting in swallowing difficulties and aspiration. The possible contribution of immunological abnormalities to these infections has not been systematically studied even though immune deficiencies have been described in patients with 22q11.2 deletion syndrome, a condition which shares remarkable clinical overlap with CHARGE syndrome. We assessed the frequency and nature of immune dysfunction in 24 children with genetically proven CHARGE syndrome. All patients, or their parents, completed a questionnaire on infectious history. Their immune system was extensively assessed through full blood counts, immunoglobulin levels, lymphocyte subpopulations, peripheral B- and T-cell differentiation, T-receptor excision circle (TREC) analysis, T-cell function, and vaccination responses. All CHARGE patients had a history of infections (often frequent), mainly otitis media and pneumonia, leading to frequent use of antibiotics and to hospital admissions. Decreased T-cell numbers were found in 12 (50%) patients, presumably caused by insufficient thymic output since TREC amounts were also diminished in CHARGE patients. Despite normal peripheral B-cell differentiation and immunoglobulin production in all patients, 83% of patients had insufficient antibody titers to one or more early childhood vaccinations. Based on our results, we recommend immunological evaluation of CHARGE patients with recurrent infections.

## Introduction

CHARGE syndrome (MIM# 214800) is a rare, multiple congenital anomaly syndrome with an estimated birth prevalence of 1 in 15,000 to 17,000 newborns [1]. The clinical diagnosis is made using criteria proposed by Blake et al. [2] or Verloes [3]. The syndrome is caused by a dominant loss-of-function mutation in, or a deletion of, the *CHD7* gene (#MIM 608892), which usually occurs *de novo* and can be found in over 90% of all children who meet the clinical diagnostic criteria. The encoding protein of *CHD7* is a member of the chromodomain helicase DNA-binding protein family that regulates the transcription of genes during embryonic development. Because of the regulating function of CHD7, haploinsufficiency of *CHD7* affects multiple organ systems, which explains the broad clinical variability seen in CHARGE syndrome. No clear genotype-phenotype correlations have been found, although variants leading to a premature stop codon are, in general, associated with a more severe phenotype than variants with a non-truncating effect (i.e. missense variants) [4].

Since Pagon et al. [5] proposed the acronym CHARGE (Coloboma of the eye, Heart defects, Atresia of the choanae, Retardation of growth and/or development, Genital abnormalities, and Ear abnormalities), new clinical features have been added to CHARGE syndrome that include cranial nerve dysfunction, absent or hypoplastic semicircular canals, anosmia, cleft lip and/or palate, and skeletal abnormalities [3,6,7]. In addition, patients with CHARGE syndrome have frequent infections including recurrent otitis media, sinusitis, and infections of the respiratory tract, which lead to morbidity and even mortality [8,9]. Deviations of the palatal and ear anatomy, as well as cranial nerve dysfunction affecting swallowing, contribute to these infections. However, the contribution of abnormalities in the immune system may be of importance because T-cell lymphopenia and thymic abnormalities have been described in individual patients with CHARGE syndrome, and these abnormalities resemble immune abnormalities seen in 22q11.2 deletion syndrome (#MIM 192430) [9]. In contrast to 22q11.2 deletion syndrome, the frequency and exact nature of the immunological abnormalities in CHARGE syndrome have so far not been studied either prospectively or systematically. In this respect, knowledge is needed to develop guidelines to optimize the care of children with CHARGE syndrome. Our aim in this study was to systematically explore the prevalence and nature of immune dysfunction in children with CHARGE syndrome.

## Patients and Methods

### Patients

Children with genetically confirmed CHARGE syndrome were recruited through the Dutch Expert Clinic for CHARGE syndrome between September 2013 and June 2014. Mutations in *CHD7* were classified as truncating (*i*.*e*. nonsense, frameshift, or deletion) or non-truncating (*i*.*e*. missense or splice-site). Healthy children, mainly siblings of CHARGE patients, were included as age-matched controls for the T-cell function assay and as a control group for the T-cell receptor excision circle (TREC) analysis. Exclusion criteria were age below 20 months or above 18 years or active infection and/or immunosuppressive therapy (e.g. steroids) at the time of the blood tests. Further exclusion criteria for healthy controls were ear-nose-throat problems in the previous two years defined as adenoidectomy, placement of tympanostomy tubes, or otitis media with effusion. Potential healthy controls with primary immune deficiencies or autoimmune disease were also excluded.

Patients, or their parents, filled in a Dutch questionnaire on infectious history (available from the authors upon request). Questions were based on international guidelines and protocols for identifying primary immune deficiency [10,11]. Additional medical information was extracted from the patient files and the database of the Dutch Expertise Centre for CHARGE syndrome. Because the thymus has not been routinely examined in CHARGE patients, information on the thymus could only be retracted from cardiac surgery reports, where available. The study was approved by the Medical Ethical Review Committee of the University Medical Centre Groningen and written informed consent was obtained from all patients, controls and/or their parents.

### Immunologic assays

Peripheral blood was obtained from all patients and healthy controls for immunological assessment. All the immunological assays we performed have been validated and are used in routine patient diagnostics. For all assays, age-matched reference values are available [12–15], except for the T-cell subpopulations of patients under the age of 5 years, TRECs, and T-cell function assay [16]. Healthy controls were used to establish age-matched reference values for the T-cell function assay and as the control group for TREC analysis. Lymphocyte populations and peripheral B- and T-cell subpopulations were also analysed in the healthy controls. Results of all assays, except for the TRECs, were assessed on the basis of the age-matched reference values.

Full blood counts were measured with an automated cell counter (Sysmex XN10/20, Kobe, Japan); serum immunoglobulin (Ig) G, IgG subclasses 1–4, IgA, and IgM were nephelometrically analysed using BNII system (Siemens AG, Munich, Germany); and serum total IgE was measured by fluoro-enzyme-immuno-assay (Phadia, Uppsala, Sweden). These assays were performed according to the manufacturer’s protocol.

IgG-specific antibodies to diphtheria toxoid and tetanus toxin were analysed at the National Institute for Public Health and the Environment (RIVM, Netherlands) using a Luminex-technology-based multiplex immunoassay developed in-house [17,18]. A protective concentration of antibody to both diphtheria and tetanus was defined as ≥0.10 IU/ml. IgG-specific antibodies to *Haemophilus influenzae* type b and to 13 types of pneumococcal polysaccharides were analysed at the laboratory of the Antonius Hospital (Nieuwegein, Netherlands). Enzyme-linked immunosorbent assay (ELISA, Binding Site, San Diego, CA, USA) was used to analyse IgG-specific antibodies to *H*. *influenzae* type b and a concentration of >1.0 mg/l was considered protective. IgG-specific antibodies to pneumococcal polysaccharides were analysed by multiplex assay [19,20]. An adequate response to pneumococcal polysaccharides was defined as an absolute level of >0.35 μg/ml in > 50% of the serotypes.

Multicolour flow cytometric phenotyping of the lymphocyte populations was performed using a FACSCanto II (Becton Dickinson, Franklin Lakes, NJ, USA) and data were analysed using FACSCanto Clinical Software version 2.4 and FACSDiva software version 7.0 (Becton Dickinson). Absolute numbers of CD3+, CD4+, and CD8+ T-cells; CD19+ B-cells; and CD16+/56+ NK-cells were measured using the MultiTest TruCount method with MultiTest reagents to CD45/3/4/8/16+56/19 (Becton Dickinson). The lyse-no-wash preparation method was performed as prescribed by the manufacturer.

Peripheral B- and T-cell differentiation was assessed by multicolour flow cytometric phenotyping of the peripheral B- and T-cell subpopulations based on the methods described by Driessen et al. [14]. The monoclonal antibodies and the gating strategy for the B- and T-cell subpopulations are discussed in S1 Appendix. Absolute numbers of the B- and T-cell subpopulations were calculated using their relative numbers and the absolute number of CD19+ B-cells or CD4+ and CD8+ T-cells. Absolute numbers of the B- and T-cell subpopulations were then compared to age-matched reference values [14,15]. Because of the heterogeneity of the peripheral T-cells, T-cell subpopulations were only considered to be decreased if both the absolute and relative numbers were lower than the age-matched reference values.

TRECs, which can be used as a reflection of thymic T-cell output [21], were assessed by the methods proposed by Hazenberg et al. [22] and Chan and Puck [23]. In brief, DNA was extracted from dry blood spots using generation DNA elution solution method (Qiagen, Hilden, Germany). Subsequently, real-time quantitative PCR for TRECs was performed with Albumin as control for DNA input. The amount of TRECs per μg DNA was calculated.

T-cell function was assessed by stimulating whole blood with five different stimuli and measuring T-cell activation (percentage CD69+ T-cells) and cytokine production (TNF-α, IFN-γ, IL-2, IL-4) as described by us earlier [16] and further described in S2 Appendix. T-cell activation and intracellular production of cytokines were determined within the CD4+ and CD8+ T-cells. The interpretation of these T-cell function tests was done by the medical immunologist (AJAL) who has extensive experience with it in our immunologic laboratory.

### Statistical analysis

We used descriptive statistics to provide summary results on individual outcomes. Fisher’s exact test was used to compare two groups of dichotomous outcomes. Student’s t test was used to compare the TRECs results of the patients with the healthy control group. In addition, the effect of age on the TRECs results was analysed with linear regression. A two-sided *p*-value smaller than 0.05 was considered as significant. The statistical software programme IBM SPSS Statistics for Windows, Version 22.0 (Armonk, NY: IBM Corp.) was used for statistical analysis. To construct graphs, GraphPad Prism for Windows, Version 5.04 (La Jolla, CA, USA) was used.

## Results

### Clinical characteristics

We initially included 27 patients and 14 healthy controls in the study. Two of the patients were later excluded because blood sampling was unsuccessful, and one patient and two healthy controls withdrew from the study. The clinical characteristics of the remaining 24 patients are presented in Table 1. The median ages of patients and controls were 8.3 (range 1.9–16.9) and 11.5 (range 5.5–17.3) years, respectively. Two-thirds of the mutations in *CHD7* in our cohort are known to lead to a premature stop in *CHD7* (truncating mutations). All but five patients fulfilled the clinical criteria for typical CHARGE syndrome as defined by Blake et al. [2] and/or Verloes [3].

**Table 1:** Clinical characteristics of 24 patients with CHARGE syndrome.

Patient	Age^1^	Sex	*CHD7* Mutation		Clinical CHARGE syndrome^2^	Infectious history			ENT procedures		Atopy	Cardiac surgery
						UAI	Pneumonia	Other	A/T	tubes		
CHD01	14.9	F	5222G>C	mis	typical	+	−	−	−	−	−	−
CHD02	8.3	M	1480C>T	non	typical	+^4^	−	++^4^	+	+	−	−
CHD04	3.8	F	8077-1G>A	splice	atypical	+	−	−	−	+	−	−
CHD05	13.8	M	2442+5G>C	splice	typical	+	−	+	−	−	−	−
CHD06	16.9	M	2442+5G>C	splice	atypical	+	−	+	+	−	−	−
CHD08	11.6	F	5405-17G>A	splice	atypical	+	−	−	−	+	−	−
CHD09	3.2	M	5405-17G>A	splice	typical	+^4^	−	−	−	+	all	−
CHD10	2.3	F	5428C>T	non	typical	−	++^3^	−	−	−	−	+
CHD11	13.5	M	8016G>A	non	typical	+^3^	+^3^	−	−	+	−	−
CHD12	5.9	F	5944_5989dup	fs	typical	+	+^3^	−	+	−	all	−
CHD13	5.8	M	7160C>A	non	typical	+^3^	−	−	−	+	−	−
CHD14	6.6	M	5241-5244del	fs	atypical	+^3^	−	++^3^	+	+	−	+
CHD15	14.4	F	5833C>T	non	typical	+^5^	−	−	+	−	all	+
CHD16	15.5	M	7650_7651del	fs	atypical	−	++^4^	−	−	−	−	−
CHD17	2.9	M	964delTT	fs	typical	+^5^	−	++^3^	−	−	ecz	−
CHD18	1.9	F	4542dup	fs	typical	−	++^4^	++^3^	−	−	−	+
CHD19	14.9	M	5405-17G>A	splice	typical	+	−	−	+	+	all	−
CHD20	8.4	M	3514_3515del	fs	typical	+	−	++^3^	−	−	−	−
CHD21	14.9	F	5181C>G	non	typical	+^3^	−	−	−	+	all; ecz	−
CHD22	3.1	F	Deletion 8q12.1q12.3	del	typical	++^3^	−	−	+	+	−	−
CHD23	8.1	M	4731delA	fs	typical	+^3^	−	−	−	−	all	−
CHD25	4.1	M	4783C>T	non	typical	−	++^4^	−	−	−	−	−
CHD26	11.8	M	5051-15T>A	splice	typical	+	−	−	−	+	−	+
CHD27	10.7	F	2572C>T	non	typical	+	++^3^	+^3^	−	+	asthma	+

-, none; +, yes; ++, yes, including hospital admission

^1^ Age in years at time of evaluation

^2^ Based on the criteria by Blake et al. [2] and/or Verloes [3]

^3^ Antibiotics given

^4^ Antibiotics given in addition to prophylactic antibiotics

^5^ Prophylatic antibiotics given

all, allergy; A/T, adenectomy and/or tonsillectomy; del, deletion; ecz, eczema; F, female; fs, frameshift; M, male; mis, missense; n, number; non, nonsense; splice, splice site; tubes, tympanostomy tubes; UAI, upper airway infection, including otitis media

All 24 patients had a history of infections (often frequent). Twenty (83%) patients had experienced upper airway infections, including otitis media in 16 (67%) patients. Seven (29%) patients had had pneumonia and eight (33%) patients had a history of other infections, including dermatomucosal infections (n = 5), gastroenteritis (n = 1), and pyelonephritis (n = 2). Ten (42%) patients had needed hospital admission for their infections, predominantly for pneumonia (n = 5). In addition, 12 (50%) patients needed placement of tympanostomy tubes because of recurrent otitis media. Antibiotics had been ever given to 17 (71%) patients and seven (29%) patients had received prophylactic antibiotics for recurrent upper airway infections or pneumonia. None of the patients had had life-threatening infections like sepsis or meningitis. Candidiasis was only seen with concomitant antibiotic use. Atopic disorders (n = 8, 33%) were mentioned as allergy (n = 6, 25%), eczema (n = 2, 8%) and asthma (n = 1, 4%). None of the patients had an autoimmune disease.

In summary, all CHARGE patients had a history of infections. Otitis media and pneumonia were the most prevalent, with prophylactic antibiotics given to 29% of patients. Eighteen patients (75%) needed hospital admission for reasons related to infectious diseases.

### Full blood count

Haemoglobin levels, numbers of erythrocytes, thrombocytes, and leukocytes (including neutrophils, lymphocytes, basophils, eosinophils, and monocytes) were normal in all 24 patients.

### Humoral immunity

Humoral immunity was evaluated by determining immunoglobulin levels and absolute numbers of peripheral B-cells and B-cell subpopulations. The levels of immunoglobulins and immunoglobulin subclasses are shown in Table 2 and were normal in all 24 patients, except for one who had a marginally decreased IgA level of 0.50 g/l (normal 0.54 g/l).

**Table 2:** Immunoglobulin levels per CHARGE patient.

Patient	Immunoglobulin levels^1^							
	IgG	IgA	IgM	IgG1	IgG2	IgG3	IgG4	IgE
CHD01	9.8 (5.2–15.6)	1.3 (0.54–3.6)	1.6 (0.13–2.4)	7.4 (3.7–12.8)	2.0 (0.85–6.1)	0.6 (0.13–1.63)	0.5 (0.023–2.3)	13 (<100)
CHD02	8.2 (5.2–15.6)	1.1 (0.54–3.6)	0.6 (0.13–2.4)	6.3 (3.7–12.8)	1.5 (0.85–6.1)	0.3 (0.13–1.63)	0.2 (0.023–2.3)	29 (<50)
CHD04	9.2 (4.3–13.4)	1.2 (0.19–2.2)	1.3 (0.21–1.8)	7.3 (3.2–12.8)	1.5 (0.52–3.4)	0.4 (0.13–1.33)	0.2 (0.012–1.58)	**20 (<10)**
CHD05	6.1 (5.2–15.6)	**0.5 (0.54–3.6)**	0.9 (0.13–2.4)	5.4 (3.7–12.8)	1.3 (0.85–6.1)	0.3 (0.13–1.63)	0.2 (0.023–2.3)	84 (<100)
CHD06	8.0 (7.0–16.0)	0.7 (0.70–4.0)	0.9 (0.40–2.3)	5.9 (3.7–12.8))	2.1 (0.85–6.1)	0.7 (0.13–1.63)	0.1 (0.023–2.3)	**420 (<100)**
CHD08	10.1 (5.2–15.6)	1.2 (0.54–3.6)	0.6 (0.13–2.4)	7.6 (3.7–12.8)	1.8 (0.85–6.1)	0.9 (0.13–1.63)	1.3 (0.023–2.3)	12 (<100)
CHD09	7.2 (4.3–13.4)	0.4 (0.19–2.2)	1.0 (0.21–1.8)	6.0 (3.2–10.0)	1.3 (0.52–3.4)	0.2 (0.13–1.33)	0.5 (0.012–1.58)	<2.0 (<10)
CHD10	7.0 (4.3–13.4)	0.5 (0.19–2.2)	1.1 (0.21–1.8)	5.8 (3.2–10.0)	1.6 (0.52–3.4)	0.3(0.13–1.33)	0.8 (0.012–1.58)	**15 (<10)**
CHD11	9.9 (5.2–15.6)	2.5 (0.54–3.6)	0.7 (0.13–2.4)	5.9 (3.7–12.8)	3.5 (0.85–6.1)	0.3 (0.13–1.63)	0.5 (0.023–2.3)	13 (<100)
CHD12	9.7 (4.3–13.4)	1.2 (0.19–2.2)	1.1 (0.21–1.8)	5.6 (3.2–10.0)	3.0 (0.52–3.4)	0.4 (0.13–1.33)	**2.2 (0.012–1.58)**	**145 (<50)**
CHD13	6.8 (4.3–13.4)	0.6 (0.19–2.2)	0.7 (0.21–1.8)	6.0 (3.2–10.0)	0.8 (0.52–3.4)	0.3 (0.13–1.33)	<0.1 (0.012–1.58)	24 (<25)
CHD14	7.0 (5.2–15.6)	1.2 (0.54–3.6)	1.3 (0.13–2.4)	5.4 (3.7–12.8)	2.1 (0.85–6.1)	0.3 (0.13–1.63)	0.3 (0.023–2.3)	7.5 (<25)
CHD15	12.3 (5.2–15.6)	2.1 (0.54–3.6)	1.2 (0.13–2.4)	8.1 (3.7–12.8)	3.4 (0.85–6.1)	0.4 (0.13–1.63)	**3.0 (0.023–2.3)**	70 (<100)
CHD16	9.4 (5.2–15.6)	1.1 (0.54–3.6)	0.5 (0.13–2.4)	7.0 (3.7–12.8)	1.8 (0.85–6.1)	0.3 (0.13–1.63)	0.8 (0.023–2.3)	22 (<100)
CHD17	11.2 (4.3–13.4)	0.8 (0.19–2.2)	1.0 (0.21–1.8)	9.2 (3.7–12.8)	1.2 (0.52–3.4)	0.4 (0.13–1.33)	0.3 (0.012–1.58)	**70 (<10)**
CHD18	8.3 (2.6–15.2)	0.7 (0.16–1.1)	0.9 (0.10–1.2)	7.3 (2.0–8.5)	1.3 (0.34–2.6)	0.5 (0.15–1.13)	<0.1 (0.011–0.79)	2.9 (<10)
CHD19	12.8 (5.2–15.6)	0.9 (0.54–3.6)	1.5 (0.13–2.4)	8.0 (3.7–12.8)	3.1 (0.85–6.1)	0.7 (0.13–1.63)	0.2 (0.023–2.3)	7.1 (<100)
CHD20	11.0 (5.2–15.6)	1.1 (0.54–3.6)	1.5 (0.13–2.4)	9.2 (3.7–12.8)	1.4 (0.85–6.1)	0.5 (0.13–1.63)	0.5 (0.023–2.3)	27 (<50)
CHD21	11.4 (5.2–15.6)	3.1 (0.54–3.6)	1.8 (0.13–2.4)	8.3 (3.7–12.8)	3.0 (0.85–6.1)	0.5 (0.13–1.63)	2.0 (0.023–2.3)	**1231 (<100)**
CHD22	6.1 (4.3–13.4)	0.5 (0.19–2.2)	0.9 (0.21–1.8)	5.2 (3.2–10.0)	1.2 (0.52–3.4)	0.2 (0.13–1.33)	<0.1 (0.012–1.58)	**190 (<10)**
CHD23	6.7 (5.2–15.6)	1.3 (0.54–3.6)	1.0 (0.13–2.4)	5.6 (3.7–12.8)	1.7 (0.85–6.1)	0.3 (0.13–1.63)	<0.1 (0.023–2.3)	26 (<50)
CHD25	12.0 (4.3–13.4)	1.4 (0.19–2.2)	0.8 (0.21–1.8)	9.2 (3.2–10.0)	1.3 (0.52–3.4)	0.4 (0.13–1.33)	0.4 (0.012–1.58)	**160 (<25)**
CHD26	12.7 (5.2–15.6)	2.3 (0.54–3.6)	2.2 (0.13–2.4)	10.6 (3.7–12.8)	0.9 (0.85–6.1)	0.5 (0.13–1.63)	0.2 (0.023–2.3)	56 (<100)
CHD27	13.3 (5.2–15.6)	1.7 (0.54–3.6)	2.3 (0.13–2.3)	10.0 (3.7–12.8)	3.3 (0.85–6.1)	1.1 (0.13–1.63)	1.0 (0.023–2.3)	**138 (<100)**

^1^ Immunoglobulin concentration in g/l, except for IgE (IE/ml). Age-matched reference values are shown in brackets [12]. Values below or above the age-matched reference values are shown in **bold**.

Absolute numbers of peripheral B-cells and B-cell subpopulations, in which relevant results were found, are shown in Table 3. Three (13%) patients had low numbers of B-cells. The absolute number of memory B-cells, consisting mainly of class-switched memory B cells and a small amount of IgM-only B-cells, is only slightly lower than the reference value in 4 of 24 (17%) CHARGE patients. Absolute numbers of all other peripheral B-cell subpopulations (transitional, naive mature, marginal zone-like, plasmablasts and CD21low B-cells) were normal or only slightly decreased in all CHARGE patients (see S1 Table).

**Table 3:** Peripheral B-cells, memory B-cells and IgM expression on class-switched memory B-cells per CHARGE patient.

Patient	B-cells^1^	Memory B-cells^1^	Class-switched memory B-cells^2^	IgM-only memory B-cells^2^	IgM expression on class-switched memory B-cells
	*CD45+ CD19+*	*CD27+ IgD-*	*CD27+ IgD- IgM-/CD27+IgD-IgG+*	*CD27+ IgD- IgG- IgM+*	*CD27+ IgD- IgM-/CD27+IgD-IgG+*
CHD01	397 (200–500)	23 (10–76)	22	1	IgM expression^3^
CHD02	420 (300–700)	18 (13–100)	18	1	N
CHD04	696 (400–1500)	52 (20–149)	46	5	IgM expression^3^
CHD05	430 (200–500)	26 (10–76)	24	2	IgM expression^3^
CHD06	360 (100–400)	19 (12–122)	19	1	IgM expression^3^
CHD08	282 (200–500)	18 (10–76)	18	0	N
CHD09	656 (400–1500)	31 (20–149)	26	5	N
CHD10	1287 (400–1500)	44 (20–149)	41	3	N
CHD11	488 (200–500)	38 (10–76)	37	1	IgM expression^3^
CHD12	562 (300–700)	76 (13–100)	74	2	N
CHD13	542 (300–700)	43 (13–100)	41	3	N
CHD14	409 (300–700)	15 (13–100)	14	1	N
CHD15	**173 (200–500)**	16 (10–76)	15	0	IgM expression^3^
CHD16	263 (200–500)	**9 (10–76)**	8	1	N
CHD17	534 (400–1500)	**19 (20–149)**	17	1	N
CHD18	**748 (900–2500)**	**8 (9–114)**	8	1	N
CHD19	273 (200–500)	15 (10–76)	14	1	IgM expression^3^
CHD20	350 (300–700)	45 (13–100)	36	9	N
CHD21	376 (200–500)	36 (10–76)	34	2	N
CHD22	1108 (400–1500)	45 (20–149)	44	1	IgM expression^3^
CHD23	337 (300–700)	23 (13–100)	21	1	N
CHD25	509 (400–1500)	**15 (20–149)**	12	2	N
CHD26	**179 (200–500)**	14 (10–76)	12	2	N
CHD27	281 (200–500)	10 (10–76)	7	3	N

^1^ Absolute numbers in cells/μl. Age-matched reference values are shown in brackets [13,14]. Values below the age-matched reference values are shown in **bold**.

^2^ No age-matched reference values available

^3^ Class-switched memory B-cells, as shown by strong IgG expression, also retaining IgM expression.

N, normal (no expression of IgM on class-switched memory B-cells).

**Fig 1:**
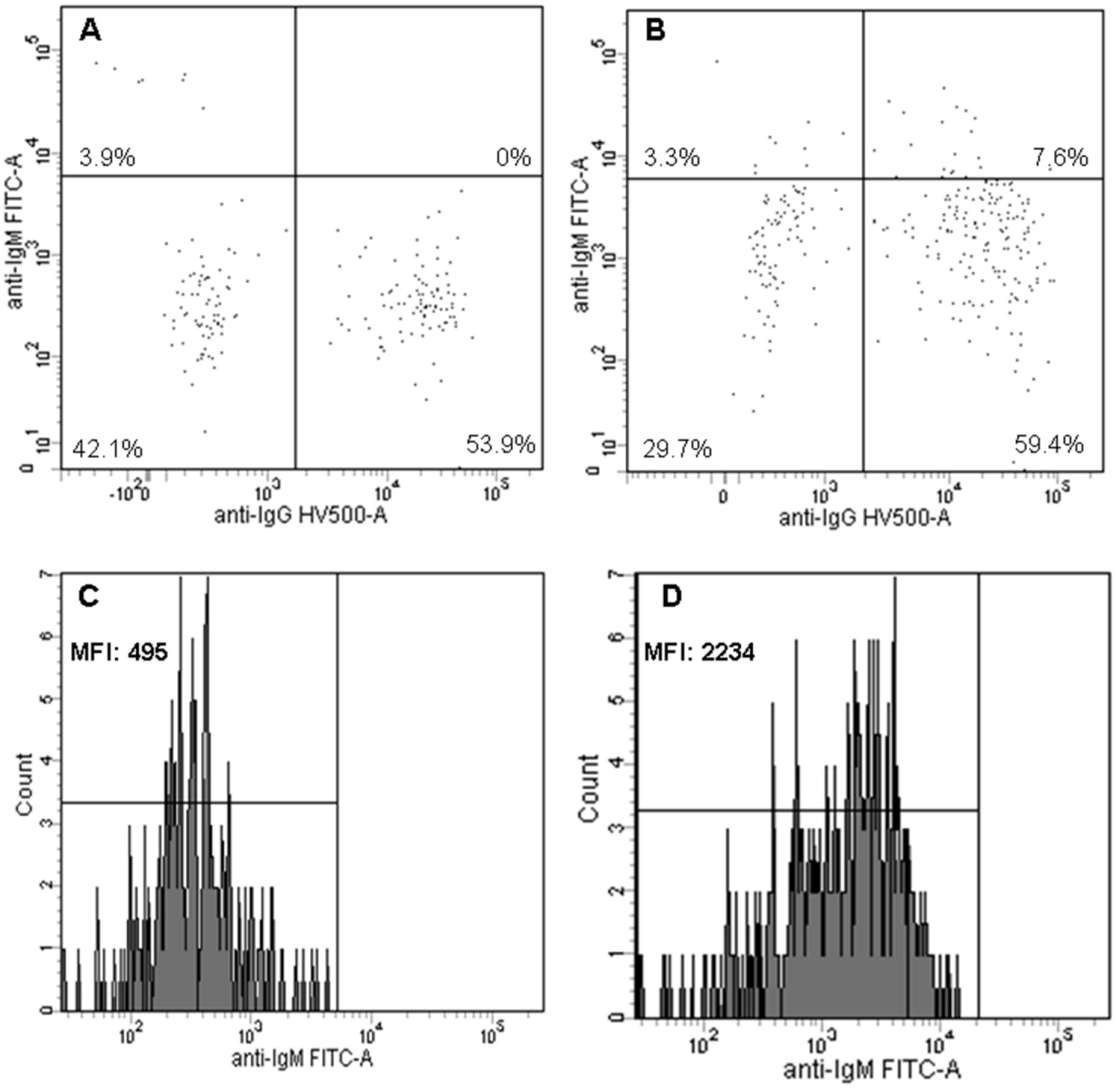
IgM and IgG expression on memory B-cells. IgM and IgG expression on memory B-cells (CD27+IgD-) of a CHARGE patient (B, D) and a simultaneously analyzed healthy control (A, C). A, B) IgG and IgM expression are used to differentiate between class-switched memory B-cells (CD27+IgD-IgM-/CD27+IgD-IgG+) and IgM-only memory B-cells (CD27+IgD-IgG-IgM++). Memory B-cells with high IgM expression and no expression of IgG are considered to be IgM-only memory B-cells (upper-left quadrant), all other memory B-cells are considered to be class-switched memory B-cells. C, D) Part of the CHARGE patients show abnormal expression of IgM on class-switched memory B-cells compared to healthy controls. MFI, mean fluorescence intensity.

Interestingly, in eight patients (33%), the memory B-cells, which clearly had undergone class-switch recombination as shown by strong IgG expression, retained IgM expression (Table 3 and Fig 1).

Thus, immunoglobulin production and peripheral B-cell differentiation were normal in our CHARGE patients, except for the abnormal expression of IgM on class-switched memory B-cells in a third of the patients.

### Cellular immunity

Cellular immunity was evaluated by determining absolute numbers of peripheral NK-cells, T-cells and T-cell subpopulations, TRECs, and T-cell function. In Table 4, the results of peripheral NK-cells, T-cells and naive T-cells are shown. Two of 24 (8%) patients had low numbers of NK-cells. Overall, 12 of 24 (50%) patients had low peripheral T-cell numbers (CD3+, CD4+ and/or CD8+ T-cells), of which five (21%) had low numbers of CD3+ T-cells, five (21%) had low numbers of CD4+ T-cells, and eleven (46%) had low numbers of CD8+ T-cells. Compared to the healthy control group, decreased CD8+ T-cell numbers were found more often in CHARGE patients (*p* = 0.031). Decreased numbers of CD4+ T-cells also occurred more often in CHARGE patients than in the healthy control group, but this difference was not significant (*p* = 0.146).

**Table 4:** Peripheral NK-cells, T-cells and naive T-cells per CHARGE patient.

Patient	NK-cells^1^	CD3+ T-cells^1^	CD4+ T-cells^1^	Naive mature CD4+ T-cells^2^	Naive mature CD4+ T-cells^2^	CD8+ T-cells^1^	Naive mature CD8+ T-cells^2^	Naive mature CD8+ T-cells^2^
	*CD16+56+*	*CD45+ CD3+*	*CD3+ CD4+*	*CD45R0- CCR7+ CD27+ CD28+*	*CD45R0- CCR7+ CD27+ CD28+*	*CD3+ CD8+*	*CD45R0- CCR7+ CD27+ CD28+*	*CD45R0- CCR7+ CD27+ CD28+*
CHD01	261 (100–700)	1628 (1000–2000)	884 (500–1300)	527 (277–796)	59.6% (42.4–66.3)	578 (300–800)	396 (205–465)	68.5% (48.8–72.9)
CHD02	584 (100–600)	1755 (1100–2800)	1209 (500–1800)	800 (515–913)	66.2% (60.2–74.6)	446 (400–1200)	261 (369–578)	58.6% (49.1–78.8)
CHD04	117 (100–700)	2010 (1400–3600)	1322 (700–2000)	1000 (n/a)	75.7% (n/a)	500 (500–1400)	405 (n/a)	81.1% (n/a)
CHD05	**88 (100–700)**	1641 (1000–2000)	1001 (500–1300)	629 (277–796)	62.8% (42.4–66.3)	452 (300–800)	273 (205–465)	60.3% (48.8–72.9)
CHD06	110 (100–400)	1110 (700–1900)	610 (400–1300)	338 (277–796)	55.4% (42.4–66.3)	250 (200–700)	147 (205–465)	58.6% (48.8–72.9)
CHD08	230 (100–700)	1285 (1000–2000)	732 (500–1300)	394 (277–796)	53.8% (42.4–66.3)	392 (300–800)	229 (205–465)	58.4% (48.8–72.9)
CHD09	467 (100–700)	1458 (1400–3600)	862 (700–2000)	553 (n/a)	64.2% (n/a)	**417 (500–1400)**	325 (n/a)	77.9% (n/a)
CHD10	957 (100–700)	2546 (1400–3600)	1488 (700–2000)	1019 (n/a)	68.5% (n/a)	813 (500–1400)	236 (n/a)	29.1% (n/a)
CHD11	234 (100–700)	1282 (1000–2000)	845 (500–1300)	488 (277–796)	57.8% (42.4–66.3)	340 (300–800)	198 (205–465)	58.4% (48.8–72.9)
CHD12	182 (100–600)	1374 (1100–2800)	915 (500–1800)	**390 (515–913)**	**42.7% (60.2–74.6)**	**357 (400–1200)**	**115 (369–578)**	**32.2% (49.1–78.8)**
CHD13	372 (100–600)	2192 (1100–2800)	1269 (500–1800)	814 (515–913)	64.2% (60.2–74.6)	652 (400–1200)	**247 (369–578)**	**37.9% (49.1–78.8)**
CHD14	330 (100–600)	**899 (1100–2800)**	**458 (500–1800)**	**215 (515–913)**	**47.0% (60.2–74.6)**	**297 (400–1200)**	**87 (369–578)**	**29.3% (49.1–78.8)**
CHD15	172 (100–700)	1110 (1000–2000)	787 (500–1300)	490 (277–796)	62.3% (42.4–66.3)	**250 (300–800)**	138 (205–465)	55.5% (48.8–72.9)
CHD16	184 (100–700)	1042 (1000–2000)	**308 (500–1300)**	**111 (277–796)**	**36.2% (42.4–66.3)**	649 (300–800)	**115 (205–465)**	**17.7% (48.8–72.9)**
CHD17	118 (100–700)	1622 (1400–3600)	894 (700–2000)	691 (n/a)	77.3% (n/a)	520 (500–1400)	79 (n/a)	71.8% (n/a)
CHD18	219 (100–1100)	**1483 (2200–5500)**	**1008 (1100–3600)**	725 (n/a)	72.0% (n/a)	**373 (500–1800)**	310 (n/a)	83.2% (n/a)
CHD19	303 (100–700)	**887 (1000–2000)**	507 (500–1300)	250 (277–796)	49.3% (42.4–66.3)	**230 (300–800)**	**94 (205–465)**	**41.0% (48.8–72.9)**
CHD20	580 (100–600)	**940 (1100–2800)**	**400 (500–1800)**	**138 (515–913)**	**34.5% (60.2–74.6)**	**290 (400–1200)**	**73 (369–578)**	**25.1% (49.1–78.8)**
CHD21	187 (100–700)	1455 (1000–2000)	917 (500–1300)	654 (277–796)	71.4% (42.4–66.3)	417 (300–800)	310 (205–465)	74.6% (48.8–72.9)
CHD22	354 (100–700)	1431 (1400–3600)	1035 (700–2000)	602 (n/a)	58.1% (n/a)	**182 (500–1400)**	102 (n/a)	56.2% (n/a)
CHD23	192 (100–600)	1239 (1100–2800)	734 (500–1800)	495 (515–913)	67.5% (60.2–74.6)	**365 (400–1200)**	265 (369–578)	72.8% (49.1–78.8)
CHD25	**98 (100–700)**	1404 (1400–3600)	896 (700–2000)	625 (n/a)	69.7% (n/a)	**422 (500–1400)**	315 (n/a)	74.7% (n/a)
CHD26	656 (100–700)	1261 (1000–2000)	775 (500–1300)	511 (277–796)	65.9% (42.4–66.3)	342 (300–800)	**143 (205–465)**	**41.9% (48.8–72.9)**
CHD27	452 (100–700)	**606 (1000–2000)**	**368 (500–1300)**	**109 (277–796)**	**29.5% (42.4–66.3)**	**169 (300–800)**	**7 (205–465)**	**4.1% (48.8–72.9)**

^1^ Absolute numbers in cells/μl. Age-matched reference values are shown in brackets [13,15]. Values below the age-matched reference values are shown in **bold**.

^2^ Absolute numbers in cells/μl and relative numbers in percentages of naive mature CD4+ or CD8+ T-cells. Age-matched reference values are shown in brackets [15]. If both the absolute and the relative numbers are below the age-matched reference values, values are shown in **bold**.

n/a, age-matched reference value not available

In the T-cell subpopulations, we primarily saw deviations in the number of naive mature T-cells. The absolute or relative numbers of other T-cell subpopulations (central memory, effector memory, terminally differentiated, activated, CD4+ regulatory, αβ, γδ and double negative αβ T-cells) were not abnormal in a relevant way (S2 Table). For seven patients, T-cell subpopulations could not be interpreted because age-matched reference values were not available. Of the other seventeen patients, five (29%) and eight (47%) patients had low numbers of naive mature CD4+ T-cells and naive mature CD8+ T-cells, respectively. Most patients with low numbers of naive mature CD4+ or CD8+ T-cells also had low numbers of total CD4+ T or CD8+ T-cells. Decreased numbers of naive mature CD4+ T-cells (*p* = 0.059) and naive mature CD8+ T-cells (*p* = 0.009) occurred more often in CHARGE patients than in the healthy control group.

Low numbers of naive mature and total T-cells can be caused by congenital thymic aplasia or hypoplasia, both of which have been described in patients with CHARGE syndrome [9]. However, low numbers of naive mature and total T-cells may also be caused by thymectomy during cardiac surgery due to congenital heart defects in CHARGE patients. Information on the thymus was available for six patients who had undergone cardiac surgery (Table 1). Two patients (CHD18 and 27) had thymectomy during cardiac surgery and had low peripheral T-cells. One patient (CHD 26) had only a partial thymectomy and had normal T-cell numbers (except for low naive CD8+ T-cells). In one patient (CHD14) thymus aplasia was confirmed during surgery, with resulting low T-cell numbers. The thymus was not mentioned in the operation reports for the other two patients (CHD10 and 15). Of the eighteen patients without cardiac surgery, eight patients (CHD09, 12, 16, 19, 20, 22, 23 and 25; 44%) had low peripheral T-cell numbers (CD3+, CD4+ and/or CD8+ T-cells).

**Fig 2:**
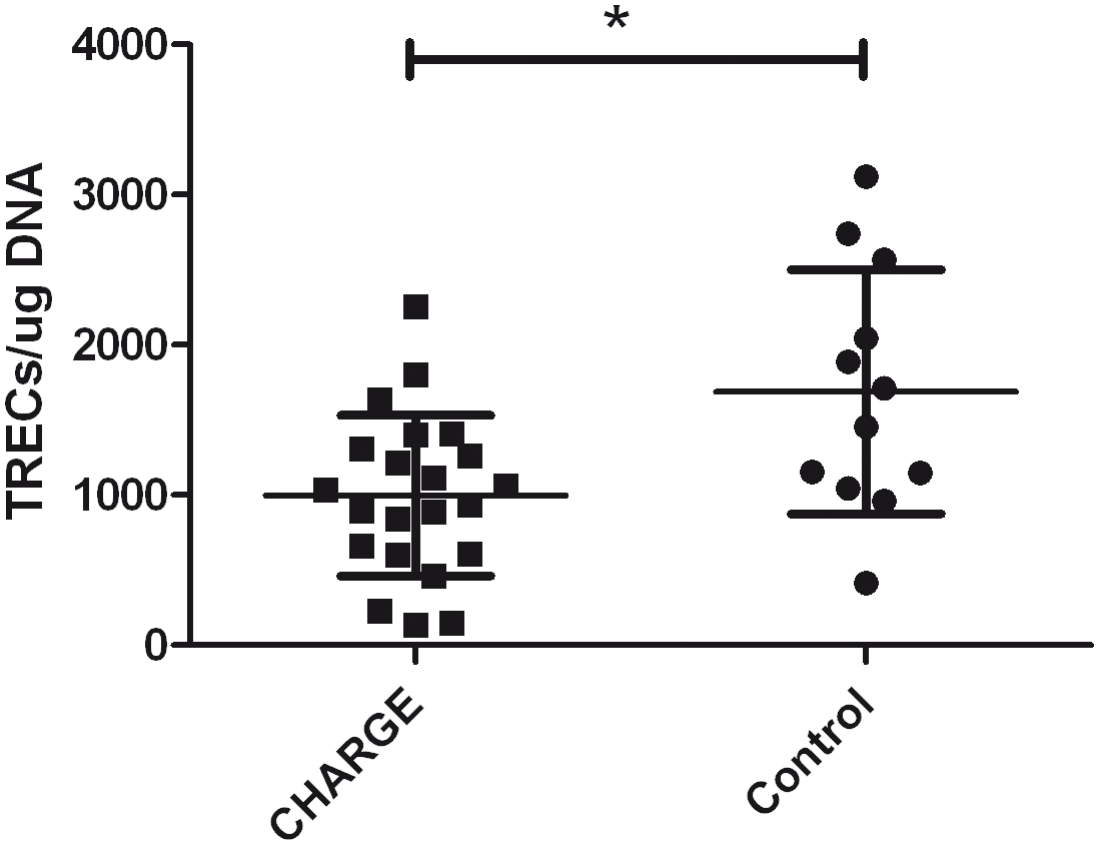
T-cell receptor excision circle (TREC) analysis. Numbers of TRECs in patients with CHARGE syndrome (n = 22) compared to healthy controls (n = 12). Error bars indicate means and standard deviations, *p = 0.005.

TREC analysis could be performed in 22 patients with CHARGE syndrome. The mean TRECs/μg DNA was 998 (SD 535), which is significantly lower than the TRECs in the healthy control group (Fig 2, mean 1688, SD 814, *p* = 0.005). To evaluate the effect of age on the amount of TRECs, a linear regression analyses was performed and showed no significant effect.

Overall, the results of all 24 patients showed sufficient T-cell activation and intracellular cytokine production after stimulation with different mitogenic and antigenic stimuli (S3 Table). Of note, 11 (46%) patients had increased T-cell responses to one or more stimuli, compared to the age-matched reference values established on the results of our 12 healthy controls. Eight of these patients had decreased percentages of naive mature T-cells and increased percentages of effector memory or terminally differentiated T-cells, which might be an explanation of the increased responses.

In summary, 50% of patients had low peripheral T-cell numbers, mainly caused by low numbers of naive mature T-cells, which is consistent with the decreased numbers of TRECs. Although the numbers of T-cells are decreased, deficiencies in T-cell function were not found with the assay we performed.

### Vaccination responses

All patients were vaccinated according to the Dutch or Belgian National Immunization Programmes [24,25], with the exception of one patient who had not been vaccinated at all (Table 5). Levels of IgG-specific antibodies to tetanus toxin were normal in all vaccinated patients. Eight of 22 (36%; assessment was unsuccessful in one patient) vaccinated patients had insufficient levels of IgG-specific antibodies to diphtheria and 15 of 23 (65%) vaccinated patients had insufficient levels of IgG-specific antibodies to *H*. *influenzae* type b. The vaccination response to pneumococcal polysaccharides could only be interpreted in 11 patients, since vaccination to pneumococcal polysaccharides was introduced into the Dutch vaccination programme in 2006. Three of 11 (27%) patients had insufficient antibodies to pneumococcal polysaccharides. Of 23 vaccinated patients, 19 (83%) had insufficient levels of IgG-specific antibodies to one or more of the vaccines we tested, while only four patients had sufficient levels of IgG-specific antibodies to all of the vaccines tested. Overall, reduced responses to one or more vaccinations, given in early childhood, are prevalent in patients with CHARGE syndrome.

**Table 5:** IgG-specific vaccine-induced antibody responses per CHARGE patient.^1^

Patient^2^	Diphtheria^3^	Tetanus^3^	Hib^4^	PPS^5^												
				PPS1	PPS3	PPS4	PPS5	PPS6A	PPS6B	PPS7F	PPS9V	PPS14	PPS18C	PPS19A	PPS19F	PPS23F
*CHD01*	**0.090**	2.06	**0.55**	**0.09**	**0.34**	**0.06**	**0.06**	0.42	**0.16**	**0.13**	**0.06**	**0.14**	3.2	8.1	7.6	1.7
CHD02	**0.089**	0.147	**0.35**	**0.33**	2.5	**0.28**	**0.07**	**0.06**	**0.09**	0.69	**0.13**	**0.07**	0.43	1.9	19	**0.27**
CHD04	**0.027**	0.155	**0.47**	**0.04**	**0.06**	**0.25**	**0.02**	**0.10**	**0.23**	**0.03**	0.56	0.54	0.59	0.51	6.8	9.8
*CHD05*	**0.070**	0.880	1.1	**0.11**	2.4	**0.04**	1	**0.09**	**0.16**	**0.13**	**0.05**	1.1	0.75	1.5	3.9	**0.04**
*CHD06*	**0.060**	7.00	**0.88**	**0.33**	14	**0.09**	**0.29**	**0.28**	3.8	0.37	**0.09**	>37	1.8	9.3	15	**0.05**
*CHD08*	0.100	7.00	**0.94**	**0.06**	**0.1**	**0.03**	**0.21**	**0.04**	**0.03**	**0.12**	**0.03**	0.86	**0.03**	1.8	15	**0.04**
CHD09	**0.066**	0.195	**0.70**	**0.10**	**0.09**	0.46	**0.12**	>23	4.4	0.54	**0.08**	**0.07**	**0.12**	**0.17**	0.80	3.9
CHD10	0.403	1.81	1.4	1.0	**0.23**	2.2	1.5	1.2	3.1	6.8	0.83	1.1	>17	**0.20**	>75	2.4
*CHD11*	**0.098**	1.35	>9.0	0.46	0.49	**0.03**	**0.11**	**0.25**	**0.26**	0.88	1.1	4.5	2.2	**0.15**	2.5	16
CHD12	1.85	1.64	**0.64**	**0.13**	4.0	>3.0	**0.070**	**0.11**	**0.26**	0.95	0.55	0.44	1.2	**0.25**	2.3	10
CHD13	0.790	9.49	**0.27**	**0.08**	**0.13**	**0.13**	**0.06**	1.6	3.9	**0.06**	0.53	9.9	1.3	5.7	>75	1.4
CHD14	1.47	4.35	**0.27**	**0.060**	1.8	**0.28**	**0.020**	2.6	3.7	>16	1.1	0.69	**0.19**	1.6	29	>21
CHD15	0.520	2.08	**0.61**	0.88	11	1.4	1.5	3.5	11	**0.26**	**0.18**	**0.24**	0.37	1.2	11	1.1
*CHD16*	**0.042**	0.101	>9.0	>8.1	**0.31**	1.6	2.1	**0.11**	**0.12**	1.0	**0.27**	9.1	**0.19**	**0.05**	1.9	**0.04**
CHD17	U	U	**0.32**	3.2	**0.16**	**0.11**	1.3	**0.04**	**0.21**	0.6	1.2	0.93	**0.21**	**0.05**	0.51	>21
CHD18	7.05	10.8	>9.0	0.7	n/a	**0.24**	0.53	n/a	0.58	1	**0.14**	2.1	**0.27**	n/a	1.3	0.54
*CHD19*	420	1.24	3.6	**0.32**	11	**0.15**	4.6	**0.06**	**0.03**	**0.31**	**0.06**	**0.18**	>17	0.96	5.2	**0.04**
*CHD20*	**0.020**	0.827	**0.30**	**0.06**	**0.19**	**0.11**	**0.02**	**0.04**	**0.06**	**0.03**	0.35	**0.08**	**0.03**	5.2	41	**0.04**
*CHD21*	0.190	0.405	**0.33**	**0.33**	**0.15**	**0.17**	1.0	**0.18**	0.52	**0.23**	4.1	1.8	**0.16**	0.66	4.7	3.0
CHD22	0.506	0.438	4.5	1.7	**0.070**	0.51	0.87	0.050	0.80	1.3	2.2	**0.16**	3.8	2.3	70	0.42
*CHD23*	1.43	1.74	**0.70**	**0.09**	0.91	**0.25**	**0.030**	1.2	6.7	1.4	0.41	0.62	5.4	2.7	59	**0.27**
CHD25	0.160	0.168	**0.72**	**0.05**	**0.09**	**0.09**	**0.01**	0.84	2.0	**0.03**	**0.23**	0.58	**0.05**	**0.08**	1.0	0.64
*CHD26*	0.311	6.20	**0.49**	1.1	2.1	**0.080**	**0.060**	**0.040**	**0.040**	**0.060**	0.41	4.7	1.6	0.70	7.7	**0.040**
*CHD27*	0.733	5.82	7.8	**0.20**	7.2	0.50	**0.28**	**0.23**	0.42	**0.30**	**0.15**	0.99	4.5	0.81	1.0	1.0

^1^ All patients were vaccinated according to the Dutch or Belgian National Immunization Programmes [24,25], with the exception of patient CHD02 who had not been vaccinated. All vaccine titers were obtained without booster or recheck.

^2^ Patients who were not vaccinated for pneumococcal polysaccharides are shown in *italic*.

^3^ Concentration of antibodies to diphtheria or tetanus in IU/ml. A concentration ≥0.10 IU/ml is considered protective. Insufficient responses (<0.10 IU/ml) are shown in **bold**.

^4^ Concentration of antibodies to *Haemophilus influenzae* type b in mg/l. A concentration >1.0 mg/l is considered protective. Insufficient responses (<1.0 mg/l) are shown in **bold**.

^5^ Concentration of antibodies to pneumococcal polysaccharides in μg/ml. An adequate response to pneumococcal polysaccharides was defined as an absolute level >0.35 μg/ml in >50% of serotypes. Insufficient responses (<0.35 μg/ml) are shown in **bold**.

Hib, *Haemophilus influenzae* type b; PPS, pneumococcal polysaccharides serotype; U, unknown

## Discussion

This is the first study to systematically and extensively explore the immune system of CHARGE patients. In our cohort of CHARGE patients, all had a history of infections (often frequent), specifically upper airway infections that often led to hospital admissions and the use of prophylactic antibiotics. Anomalies in the upper airway (atresia of choanae, abnormal outer and inner ear anatomy) contribute to patient susceptibility to infections and extend infection duration by impeding drainage or clearance of infectious debris. It is, however important, to know whether immunological abnormalities contribute to the frequency and complicate the severity of infections in order to optimize the management of care in these patients.

No abnormalities were found in routine diagnostics (full blood count), but with detailed immunologic assays, we found T-cell lymphopenia in 50% of patients, mainly caused by low numbers of naive mature T-cells. Our finding is comparable with the results of a retrospective study by Jyonouchi et al. [8], who found overall T-cell lymphopenia in four of nine (44%) CHARGE patients.

Notably, we found low T-cell numbers in 44% of patients who had not undergone cardiac surgery and therefore should have an “intact” thymus. Congenital dysmorphology or dysfunction of the thymus might be the underlying cause of T-cell lymphopenia and this is supported by our finding of diminished TREC numbers in the patients. Little is known about thymic abnormalities in CHARGE patients from the literature. Thymic anomalies have been reported in foetuses with confirmed *CHD7* mutations [26] and were reported in 16 of 36 (44%) patients with a proven mutation in *CHD7* [9]. Unfortunately, there is no specific information on the thymus for 18 of 24 patients in our CHARGE cohort.

Evidence for the role of *CHD7* and *TBX1*, the causative gene of 22q11.2 deletion syndrome, has been shown in the embryonic development of the thymus in animal models. Both genes are expressed in the pharyngeal arches which contain precursors of thymic stromal cells [27,28]. Bi-directional molecular interaction between thymic epthelial cells and T-cell progenitor cells is critical for the complete morphological and functional maturation of both cell compartments [27]. Thus, abnormal thymic development presumably not only affects the level of T-cell output, but could also affect the function of T-cells. For example, the T-cell receptor repertoire [29] and the development of natural regulatory T-cells [30] have been shown to be affected in patients with 22q11.2 deletion syndrome. Although we found normal T-cell responses with our T-cell function assay, we cannot exclude subtle dysfunctions in more complex T-cell function, such as the delicate interaction between T-cells and B-cells.

Peripheral B-cell and NK-cell numbers were normal in almost all patients, comparable to former reports [9]. However, hypogammaglobulinaemia was found in 61% of CHARGE patients in former case reports [9], while in our study the immunoglobulin levels were normal in all patients. Publication bias needs to be taken in consideration for the higher percentages found in case reports. Actually, our results were more comparable with those from 22q11.2 deletion syndrome, where hypogammaglobulinaemia was found in only 6% of a large cohort of 855 patients [31]. Although peripheral B-cell differentiation was normal, a third of the CHARGE patients had class-switched memory B-cell retaining IgM expression. We had not anticipated on this finding in our methods by including isotype controls, which limits the interpretation of these data. Nonetheless, these cells may indicate impaired class-switch recombination and memory B-cell formation in CHARGE patients. To our knowledge, these phenotypically abnormal class-switched memory B-cells have not been reported before, but lower numbers of class-switched memory B-cells have been found in adults with 22q11.2 deletion syndrome [32]. We can speculate that the formation of fully functional class-switched memory B-cells in CHARGE patients is impeded due to insufficient T-cell help during the class-switch recombination process, leading to diminished production of specific antibodies. However, our data is insufficient to fully support this tentative hypothesis which links our findings in the peripheral T-cell populations with the humoral abnormalities. More research is needed on memory B-cell formation and function in CHARGE patients.

Specific antibodies to one or more vaccines given in childhood were insufficient in 83% of patients, specifically to diphtheria and *H*. *influenzae* type b vaccines. In the literature, vaccination responses in CHARGE syndrome are only described in case reports and in one retrospective study. Reduced responses to diphtheria (n = 3), tetanus (n = 4), *H*. *influenzae* type b (n = 2), and pneumococcal polysaccharides (n = 1) have been reported [8,33,34]. Although protective levels of specific antibodies decrease over time, this waning seems to occur at an earlier age in patients with CHARGE syndrome (median age 14.7 years) compared with the general population (30–40 years of age) [35,36].

We studied the largest cohort of well-defined CHARGE patients with confirmed *CHD7* mutations so far but, of course, statistical analysis on only 24 patients limits the interpretation of our results. What we can state is that immunological abnormalities are often seen in patients with CHARGE syndrome. We hypothesize that abnormal thymic development leads to diminished numbers of T-cells that may also be impaired in more subtle functions as activating B-cells to differentiate into fully functional class-switched memory B-cells. Incomplete class-switched memory B-cell formation can be an explanation for the insufficient responses to vaccines in our CHARGE patients due to poor humoral memory.

The high prevalence of immunological abnormalities combined with the frequent occurrence of infections demonstrates the need for more research in a larger cohort to extend the analysis of correlations between clinical data and immunological laboratory results, and to confirm some of our immunological findings. With such data, evidence-based guidelines can be developed for the timely diagnosis of immune dysfunctions based on clinical symptoms, which will help protect these children from excess morbidity and mortality due to infections. Nonetheless, based on the results of this study, we would recommend performing specialised immunologic assays (B- and T-cell numbers and vaccination responses) in patients with persistent infections who need prophylactic antibiotics, since the immune abnormalities we found will not be apparent with routine diagnostics. Considering the high prevalence of reduced antibody responses, it may be worthwhile to give these patients booster vaccinations and recheck the antibody responses. Firstly because *H*. *influenza* and pneumococcal infections are highly prevalent in otitis media [37], and secondly to determine if CHARGE patients have a primary lack of response to some vaccines or if they have poor humoral memory.

## Supporting Information

S1 Appendix Monoclonal antibodies and gating strategy for the B- and T-cell subpopulations. (DOC)

S2 Appendix Stimulation, fluorescent barcoding, and monoclonal antibodies in the T-cell function assay. (DOCX)

S1 Dataset Data of the T-cell receptor excision circle analysis. (XLSX)

S1 Table Peripheral B-cell subpopulations per CHARGE patient. (DOC)

S2 Table Peripheral T-cells subpopulations per CHARGE patient. (DOC)

S3 Table T-cell function assay per CHARGE patient. (XLS)
