# Supplementary material for: Immune Dysfunction in Children with CHARGE Syndrome: A Cross-Sectional Study
Source: PLoS One. 2015 Nov 6;10(11):e0142350. doi: 10.1371/journal.pone.0142350 (PMC4636349; doi:10.1371/journal.pone.0142350)
Supplement: S1 Appendix — (DOC) [file pone.0142350.s001.doc]

**S1 Appendix. Monoclonal antibodies and gating strategy for the B- and T-cell subpopulations.**

The following monoclonal antibodies were used: CD19/CD3-PerCP-Cy5.5 (SJ25C1/SK3); CD27/CD25-APC (L128/2A3), CD38/CD8-APC-H7 (HB7/SK1), IgD/CD27/CD127/TCRγδ-PE (IA6-2/L128/HIL-7R-M21/11F2); IgM/CD45R0/TCRαβ-FITC (Polyclonal/UCHL1/WT31); IgG-BV510 (G18-145), CD21/CD4-Horizon V450 (B-ly4/RPA-T4); CD24/CD28/HLA-DR-PE-c7 (ML5/CD28.2/L243); and CCR7-Alexa Fluor 647 (150503). All reagents were purchased from Becton Dickinson, except for IgM-FITC (IQProducts, Groningen , Netherlands). Within the B-cells (CD19+), transitional (CD27- CD24++ CD38+), naive mature (CD27- IgD+), marginal zone-like (CD27+ IgD+), memory (CD27+ IgD-), class-switched memory (CD27+ IgD- IgM-/CD27+IgD-IgG+), IgM-only memory (CD27+ IgD- IgG- IgM++), CD27- memory (CD27- IgD-), plasmablasts (CD27++ IgM- CD24- CD38++), and CD21low (CD21low CD38-) B-cell subpopulations were distinguished. Within the CD4+ and CD8+ T cells, naïve (CD45R0- CCR7+ CD27+ CD28+), central memory (CD45R0+ CCR7+), effector memory (CD45R0+ CCR7-), terminally differentiated (CD45R0- CCR7-/CD45R0- CCR7+ CD28-), and activated (HLA-DR+) T-cell subpopulations were distinguished. Furthermore, CD4+ regulatory (CD25+ CD127-), double negative αβ (CD3+ CD4- CD8- TCRαβ+), and γδ (CD3+ TCRγδ+) T-cells were distinguished.
