# Supplementary material for: Immune Dysfunction in Children with CHARGE Syndrome: A Cross-Sectional Study
Source: PLoS One. 2015 Nov 6;10(11):e0142350. doi: 10.1371/journal.pone.0142350 (PMC4636349; doi:10.1371/journal.pone.0142350)
Supplement: S2 Appendix — (DOCX) [file pone.0142350.s002.docx]

**S2 Appendix. Stimulation, fluorescent barcoding, and monoclonal antibodies in the T-cell function assay.**

Heparinised whole blood was stimulated with either 10 μg/ml purified protein derivative (PPD; Statens serum institute, Copenhagen, Denmark), 5 µg/ml Staphylococcal enterotoxin B (SEB; Sigma, Deisenhofen, Germany), 100 µl anti-CD3 (in house stock of WT32, 1:10 diluted), 15 Lf/ml Tetanus toxoid (TT; Statens serum institute), or 5 µg/ml phytohemagglutinin (PHA; Remel, Lenexa, KS, USA). Unstimulated blood was used as negative control. To all samples, except PHA and SEB stimulated samples, 2 µl CD28/CD49d (1 mg/ml) was added. After 2 hours of incubation at 37ºC, 2 µl Brefaldin A (1 mg/ml) was added, and the samples were incubated for another 18-22 hours. After incubation and lysation, samples were stained with different concentrations of Pacific Orange and/or Pacific Blue for fluorescent barcoding (Invitrogen Carlsbad, CA, USA). Subsequently, samples were stained with the following monoclonal antibodies: CD3-FITC (UCHT1), CD4-PE-Cy7 (SK3), CD8-PerCP (SK1), CD69-APC-Cy7 (FN50), IFN-γ-PE (B27), TNF-α-APC (Mab11), IL-2-PE (MQ1-17H12), and IL-4-APC (8D4-8). IFN-γ-PE and IL-2-PE were stained in separate tubes. The same applies for TNF-α-APC and IL-4-APC. All reagents were purchased from Becton Dickinson.
