# Supplementary material for: Immune Dysfunction in Children with CHARGE Syndrome: A Cross-Sectional Study
Source: PLoS One. 2015 Nov 6;10(11):e0142350. doi: 10.1371/journal.pone.0142350 (PMC4636349; doi:10.1371/journal.pone.0142350)
Supplement: S1 Table — (DOC) [file pone.0142350.s004.doc]

**S1 Table. Peripheral B-cell subpopulations per CHARGE patient.**

| **Patient** | **Transitional B-cells^1^** | **Naive mature B-cells^1^** | **Marginal zone-like B-cells^1^** | **CD27- memory^1^** | **Plasmablasts^1^** | **CD21low^2^** |
| --- | --- | --- | --- | --- | --- | --- |
|  | *CD27- CD24++ CD38+* | *CD27- IgD+* | *CD27+ IgD+* | *CD27- IgD-* | *CD27++ IgM- CD24- CD38++* | *CD21low CD38-* |
| CHD01 | 59 (4-108) | 251 (87-390) | 47 (7-90) | 6 (n/a) | 1 (0.5-20) | 3.4% (<10%) |
| CHD02 | 50 (11-77) | 284 (111-486) | 42 (15-88) | 13 (n/a) | **0 (1-15)** | 3.5% (<10%) |
| CHD04 | 116 (24-333) | 401 (170-1691) | 72 (16-226) | 19 (n/a) | 5 (n/a) | 5.0% (<10%) |
| CHD05 | 40 (4-108) | 289 (87-390) | 55 (7-90) | 10 (n/a) | **0 (0.5-20)** | 4.7% (<10%) |
| CHD06 | 23 (3-50) | 278 (57-447) | 25 (9-88) | 7 (n/a) | **0 (1-23)** | 3.1% (<10%) |
| CHD08 | 55 (4-108) | 170 (87-390) | 20 (7-90) | 4 (n/a) | 1 (0.5-20) | 1.8% (<10%) |
| CHD09 | 109 (24-333) | 423 (170-1691) | 49 (16-226) | 16 (n/a) | 1 (n/a) | 3.9% (<10%) |
| CHD10 | 239 (24-333) | 859 (170-1691) | 75 (16-226) | 17 (n/a) | 5 (n/a) | 2.7% (<10%) |
| CHD11 | 122 (4-108) | 247 (87-390) | 44 (7-90) | 9 (n/a) | 6 (0.5-20) | 2.8% (<10%) |
| CHD12 | 83 (11-77) | 306 (111-486) | 56 (15-88) | 22 (n/a) | 6 (1-15) | 7.2% (<10%) |
| CHD13 | 65 (11-77) | 293 (111-486) | 98 (15-88) | 12 (n/a) | 5 (1-15) | 6.6% (<10%) |
| CHD14 | 64 (11-77) | 263 (111-486) | 41 (15-88) | 9 (n/a) | 3 (1-15) | 5.0% (<10%) |
| CHD15 | 15 (4-108) | 105 (87-390) | 16 (7-90) | 3 (n/a) | 6 (0.5-20) | 3.6% (<10%) |
| CHD16 | 20 (4-108) | 210 (87-390) | 14 (7-90) | 4 (n/a) | 1 (0.5-20) | 3.1% (<10%) |
| CHD17 | 142 (24-333) | 279 (170-1691) | 56 (16-226) | 10 (n/a) | 3 (n/a) | 4.4% (<10%) |
| CHD18 | 211 (38-551) | 441 (322-1991) | 38 (23-195) | 4 (n/a) | 1 (n/a) | 3.8% (<10%) |
| CHD19 | 65 (4-108) | 152 (87-390) | 19 (7-90) | 4 (n/a) | 2 (0.5-20) | 6.7% (<10%) |
| CHD20 | 27 (11-77) | 166 (111-486) | 75 (15-88) | 20 (n/a) | 2 (1-15) | 9.4% (<10%) |
| CHD21 | 43 (4-108) | 227 (87-390) | 44 (7-90) | 12 (n/a) | 2 (0.5-20) | 3.6% (<10%) |
| CHD22 | 177 (24-333) | 700 (170-1691) | 127 (16-226) | 14 (n/a) | 4 (n/a) | 4.7% (<10%) |
| CHD23 | 64 (11-77) | 183 (111-486) | 30 (15-88) | 12 (n/a) | 4 (1-15) | 7.2% (<10%) |
| CHD25 | 118 (24-333) | 311 (170-1691) | 44 (16-226) | 4 (n/a) | 2 (n/a) | 2.7% (<10%) |
| CHD26 | 33 (4-108) | 105 (87-390) | 10 (7-90) | 6 (n/a) | 1 (0.5-20) | 8.9% (<10%) |
| CHD27 | 67 (4-108) | 172 (87-390) | 18 (7-90) | 5 (n/a) | **0 (0.5-20)** | 9.6% (<10%) |

^1^ Absolute numbers in cell/µL. Age-matched reference values are shown in brackets [14]. Values below the age-matched reference values are shown in **bold**.

^2^ Relative numbers in percentages within B-cells. Age-matched reference values are shown in brackets. Values above the age-matched reference values are shown in **bold**.

n/a, age-matched reference value not available
