# Supplementary material for: Immune Dysfunction in Children with CHARGE Syndrome: A Cross-Sectional Study
Source: PLoS One. 2015 Nov 6;10(11):e0142350. doi: 10.1371/journal.pone.0142350 (PMC4636349; doi:10.1371/journal.pone.0142350)
Supplement: S2 Table — (DOC) [file pone.0142350.s005.doc]

**S2 Table. Peripheral T-cells subpopulations per CHARGE patient.**

| **Patient** | **Central memory CD4+ T-cells^1^** | **Central memory CD4+ T-cells^2^** | **Effector memory CD4+ T-cells^1^** | **Effector memory CD4+ T-cells^2^** | **Terminally differentiated CD4+ T-cells^1^** | **Terminally differentiated CD4+ T-cells^2^** | **Activated CD4+ T-cells^2^** |
| --- | --- | --- | --- | --- | --- | --- | --- |
|  | *CD45R0+ CCR7+* | *CD45R0+ CCR7+* | *CD45R0+ CCR7-* | *CD45R0+ CCR7-* | *CD45R0- CCR7-/CD45R0- CCR7+ CD28-* | *CD45R0- CCR7-/CD45R0- CCR7+ CD28-* | *HLA-DR+* |
| CHD01 | 218 (127-270) | 24.7% (13.7-31.9) | 106 (83-206) | 12.0% (11.8-26.2) | 28 (14-76) | 3.2% (1.4-7.8) | 3.0% (<5%) |
| CHD02 | 227 (151-249) | 18.8% (14.2-22.8) | 135 (82-166) | 11.2% (7.4-17.2) | 45 (16-67) | 3.7% (1.4-5.4) | 4.5% (<5%) |
| CHD04 | 239 (n/a) | 18.1% (n/a) | 57 (n/a) | 4.3% (n/a) | 22 (n/a) | 1.7% (n/a) | 2.0% (<5%) |
| CHD05 | 238 (127-270) | 23.8% (13.7-31.9) | 115 (83-206) | **11.5% (11.8-26.2)** | 18 (14-76) | 1.8% (1.4-7.8) | 3.9% (<5%) |
| CHD06 | 127 (127-270) | 20.8% (13.7-31.9) | 114 (83-206) | 18.7% (11.8-26.2) | 31 (14-76) | 5.0% (1.4-7.8) | **6.6% (<5%)** |
| CHD08 | 182 (127-270) | 24.9% (13.7-31.9) | 143 (83-206) | 19.5% (11.8-26.2) | **12 (14-76)** | 1.7% (1.4-7.8) | **6.3% (<5%)** |
| CHD09 | 234 (n/a) | 27.2% (n/a) | 52 (n/a) | 6.0% (n/a) | 22 (n/a) | 2.5% (n/a) | 3.1% (<5%) |
| CHD10 | 277 (n/a) | 18.6% (n/a) | 113 (n/a) | 7.6% (n/a) | 70 (n/a) | 4.7% (n/a) | 3.0% (<5%) |
| CHD11 | 193 (127-270) | 22.8% (13.7-31.9) | 145 (83-206) | 17.2% (11.8-26.2) | 19 (14-76) | 2.2% (1.4-7.8) | 4.1% (<5%) |
| CHD12 | **259 (151-249)** | **28.3% (14.2-22.8)** | **237 (82-166)** | **25.9% (7.4-17.2)** | 28 (16-67) | 3.1% (1.4-5.4) | **6.0% (<5%)** |
| CHD13 | **294 (151-249)** | **23.2% (14.2-22.8)** | 138 (82-166) | 10.9% (7.4-17.2) | 19 (16-67) | 1.5% (1.4-5.4) | 2.3% (<5%) |
| CHD14 | **123 (151-249)** | **26.8% (14.2-22.8)** | 90 (82-166) | **19.7% (7.4-17.2)** | 28 (16-67) | **6.1% (1.4-5.4)** | 4.5% (<5%) |
| CHD15 | 182 (127-270) | 23.1% (13.7-31.9) | 95 (83-206) | 12.1% (11.8-26.2) | 19 (14-76) | 2.4% (1.4-7.8) | 3.0% (<5%) |
| CHD16 | **107 (127-270)** | **34.7% (13.7-31.9)** | **79 (83-206)** | 25.6% (11.8-26.2) | **10 (14-76)** | 3.4% (1.4-7.8) | **7.3% (<5%)** |
| CHD17 | 149 (n/a) | 16.7% (n/a) | 34 (n/a) | 3.8% (n/a) | 18 (n/a) | 2.0% (n/a) | 2.7% (<5%) |
| CHD18 | 195 (n/a) | 19.4% (n/a) | 64 (n/a) | 6.4% (n/a) | 21 (n/a) | 2.1% (n/a) | 3.0% (<5%) |
| CHD19 | 159 (127-270) | 31.3% (13.7-31.9) | 93 (83-206) | 18.3% (11.8-26.2) | **6 (14-76)** | **1.1% (1.4-7.8)** | **6.4% (<5%)** |
| CHD20 | 151 (151-249) | **37.8% (14.2-22.8)** | 99 (82-166) | **24.7% (7.4-17.2)** | **11 (16-67)** | 2.8% (1.4-5.4) | **5.2% (<5%)** |
| CHD21 | 176 (127-270) | 19.2% (13.7-31.9) | **59 (83-206)** | **6.4% (11.8-26.2)** | 25 (14-76) | 2.7% (1.4-7.8) | 2.2% (<5%) |
| CHD22 | 276 (n/a) | 26.6% (n/a) | 121 (n/a) | 11.7% (n/a) | 24 (n/a) | 2.3% (n/a) | **6.3% (<5%)** |
| CHD23 | 165 (151-249) | 22.5% (14.2-22.8) | **59 (82-166)** | 8.0% (7.4-17.2) | **14 (16-67)** | 1.9% (1.4-5.4) | 2.9% (<5%) |
| CHD25 | 210 (n/a) | 23.4% (n/a) | 47 (n/a) | 5.3% (n/a) | 13 (n/a) | 1.5% (n/a) | 1.6% (<5%) |
| CHD26 | **102 (127-270)** | **13.2% (13.7-31.9)** | **66 (83-206)** | **8.5% (11.8-26.2)** | **92 (14-76)** | **11.9% (1.4-7.8)** | 2.2% (<5%) |
| CHD27 | 147 (127-270) | **40.0% (13.7-31.9)** | 87 (83-206) | 23.7% (11.8-26.2) | 23 (14-76) | 6.2% (1.4-7.8) | 4.2% (<5%) |

| **Patient** | **Central memory CD8+ T-cells^1^** | **Central memory CD8+ T-cells^2^** | **Effector memory CD8+ T-cells^1^** | **Effector memory CD8+ T-cells^2^** | **Terminally differentiated CD8+ T-cells^1^** | **Terminally differentiated CD8+ T-cells^2^** | **Activated CD8+ T-cells^2^** |
| --- | --- | --- | --- | --- | --- | --- | --- |
|  | *CD45R0+ CCR7+* | *CD45R0+ CCR7+* | *CD45R0+ CCR7-* | *CD45R0+ CCR7-* | *CD45R0- CCR7-/CD45R0- CCR7+ CD28-* | *CD45R0- CCR7-/CD45R0- CCR7+ CD28-* | *HLA-DR+* |
| CHD01 | 15 (11-60) | 2.6% (1.9-11.4) | 72 (35-111) | 12.5% (5.3-18.6) | 94 (68-207) | 16.2% (13.0-30.4) | 4.3% (<11%) |
| CHD02 | 21 (6-63) | 4.7% (0.9-9.3) | 82 (26-114) | **18.5% (3.8-13.8)** | **81 (82-246)** | 18.2% (11.0-32.6) | **16.0% (<11%)** |
| CHD04 | 10 (n/a) | 2.0% (n/a) | 28 (n/a) | 5.7% (n/a) | 56 (n/a) | 11.3% (n/a) | 2.0% (<11%) |
| CHD05 | **10 (11-60)** | 2.3% (1.9-11.4) | 96 (35-111) | **21.2% (5.3-18.6)** | 73 (68-207) | 16.2% (13.0-30.4) | 5.2% (<11%) |
| CHD06 | **10 (11-60)** | 4.0% (1.9-11.4) | 65 (35-111) | **25.8% (5.3-18.6)** | **29 (68-207)** | 11.5% (13.0-30.4) | **11.8% (<11%)** |
| CHD08 | 11 (11-60) | 2.8% (1.9-11.4) | 80 (35-111) | **20.4% (5.3-18.6)** | 72 (68-207) | 18.3% (13.0-30.4) | 6.8% (<11%) |
| CHD09 | 9 (n/a) | 2.1% (n/a) | 28 (n/a) | 6.8% (n/a) | 55 (n/a) | 13.1% (n/a) | 2.5% (<11%) |
| CHD10 | 13 (n/a) | 1.6% (n/a) | 89 (n/a) | 11.0% (n/a) | 469 (n/a) | 57.7% (n/a) | 7.0% (<11%) |
| CHD11 | 18 (11-60) | 5.2% (1.9-11.4) | 73 (35-111) | **21.5% (5.3-18.6)** | **51 (68-207)** | 15.0% (13.0-30.4) | 6.4% (<11%) |
| CHD12 | 15 (6-63) | 4.2% (0.9-9.3) | **121 (26-114)** | **33.9% (3.8-13.8)** | 105 (82-246) | 29.5% (11.0-32.6) | 8.6% (<11%) |
| CHD13 | 33 (6-63) | 5.0% (0.9-9.3) | **216 (26-114)** | **33.1% (3.8-13.8)** | 156 (82-246) | 24.0% (11.0-32.6) | **12.0% (<11%)** |
| CHD14 | 14 (6-63) | 4.6% (0.9-9.3) | 73 (26-114) | **24.8% (3.8-13.8)** | 121 (82-246) | **40.9% (11.0-32.6)** | **18.4% (<11%)** |
| CHD15 | 11 (11-60) | 4.4% (1.9-11.4) | 74 (35-111) | **29.7% (5.3-18.6)** | **26 (68-207)** | **10.5% (13.0-30.4)** | 4.8% (<11%) |
| CHD16 | **7 (11-60)** | **1.1% (1.9-11.4)** | **136 (35-111)** | **21.0% (5.3-18.6)** | **389 (68-207)** | **60.0% (13.0-30.4)** | 10.6% (<11%) |
| CHD17 | 8 (n/a) | 1.9% (n/a) | 89 (n/a) | 12.4% (n/a) | 16 (n/a) | 14.0% (n/a) | 7.0% (<11%) |
| CHD18 | 6 (n/a) | 1.7% (n/a) | 17 (n/a) | 4.6% (n/a) | 39 (n/a) | 10.4% (n/a) | 4.5% (<11%) |
| CHD19 | **10 (11-60)** | 4.3% (1.9-11.4) | 106 (35-111) | **46.3% (5.3-18.6)** | **19 (68-207)** | **8.4% (13.0-30.4)** | **18.8% (<11%)** |
| CHD20 | 9 (6-63) | 3.0% (0.9-9.3) | **123 (26-114)** | **42.5% (3.8-13.8)** | 85 (82-246) | 29.4% (11.0-32.6) | 7.5% (<11%) |
| CHD21 | **7 (11-60)** | **1.6% (1.9-11.4)** | **26 (35-111)** | 6.3% (5.3-18.6) | 72 (68-207) | 17.4% (13.0-30.4) | 2.1% (<11%) |
| CHD22 | 5 (n/a) | 2.6% (n/a) | 29 (n/a) | 15.9% (n/a) | 46 (n/a) | 25.0% (n/a) | 10.2% (<11%) |
| CHD23 | 13 (6-63) | 3.5% (0.9-9.3) | 52 (26-114) | **14.4% (3.8-13.8)** | **33 (82-246)** | **9.2% (11.0-32.6)** | 4.4% (<11%) |
| CHD25 | 14 (n/a) | 3.4% (n/a) | 40 (n/a) | 9.4% (n/a) | 52 (n/a) | 12.4% (n/a) | 1.9% (<11%) |
| CHD26 | 12 (11-60) | 3.4% (1.9-11.4) | 76 (35-111) | **22.1% (5.3-18.6)** | 110 (68-207) | **32.3% (13.0-30.4)** | 4.2% (<11%) |
| CHD27 | **2 (11-60)** | **1.2% (1.9-11.4)** | 83 (35-111) | **48.9% (5.3-18.6)** | 77 (68-207) | **45.7% (13.0-30.4)** | 5.7% (<11%) |

| **Patient** | **CD4+ regulatory T-cells^2^** | **αβ T-cells^2^** | **γδ T-cells^2^** | **Double negative αβ T-cells^2^** |
| --- | --- | --- | --- | --- |
|  | *CD25+ CD127-* | *CD3+ TCRαβ+* | *CD3+ TCRγδ+* | *CD3+ CD4- CD8- TCRαβ+* |
| CHD01 | 8.6% (3-10%) | 89.2% (81-98%) | 10.4% (1-18%) | 2.3% (<2.5%) |
| CHD02 | 4.0% (3-10%) | 95.6% (81-98%) | 4.0% (1-18%) | 2.2% (<2.5%) |
| CHD04 | 8.4% (3-10%) | 92.1% (81-98%) | 7.5% (1-18%) | 1.7% (<2.5%) |
| CHD05 | 6.6% (3-10%) | 84.1% (81-98%) | 15.1% (1-18%) | 2.0% (<2.5%) |
| CHD06 | 8.3% (3-10%) | **67.5% (81-98%)** | **32.0% (1-18%)** | 1.8% (<2.5%) |
| CHD08 | 5.8% (3-10%) | 88.8% (81-98%) | 10.9% (1-18%) | **3.0% (<2.5%)** |
| CHD09 | 7.1% (3-10%) | 91.7% (81-98%) | 7.3% (1-18%) | **3.2% (<2.5%)** |
| CHD10 | 8.0% (3-10%) | 96.2% (81-98%) | 2.7% (1-18%) | **5.4% (<2.5%)** |
| CHD11 | 9.0% (3-10%) | 92.7% (81-98%) | 6.9% (1-18%) | 1.6% (<2.5%) |
| CHD12 | 10.0% (3-10%) | 93.5% (81-98%) | 5.5% (1-18%) | 1.6% (<2.5%) |
| CHD13 | 6.3% (3-10%) | 87.6% (81-98%) | 11.9% (1-18%) | 1.2% (<2.5%) |
| CHD14 | 7.8% (3-10%) | 84.2% (81-98%) | 15.7% (1-18%) | 1.6% (<2.5%) |
| CHD15 | 8.0% (3-10%) | 93.4% (81-98%) | 6.3% (1-18%) | 1.4% (<2.5%) |
| CHD16 | 4.3% (3-10%) | 90.6% (81-98%) | 8.3% (1-18%) | **4.5% (<2.5%)** |
| CHD17 | 7.3% (3-10%) | 83.9% (81-98%) | 15.9% (1-18%) | 1.3% (<2.5%) |
| CHD18 | 5.4% (3-10%) | 95.2% (81-98%) | 4.2% (1-18%) | **4.5% (<2.5%)** |
| CHD19 | 7.7% (3-10%) | 84.1% (81-98%) | 14.7% (1-18%) | 1.7% (<2.5%) |
| CHD20 | 9.4% (3-10%) | **73.6% (81-98%)** | **26.1% (1-18%)** | 2.2% (<2.5%) |
| CHD21 | 6.5% (3-10%) | 90.1% (81-98%) | 8.5% (1-18%) | **2.6% (<2.5%)** |
| CHD22 | 7.0% (3-10%) | 90.1% (81-98%) | 9.7% (1-18%) | 2.4% (<2.5%) |
| CHD23 | 8.5% (3-10%) | 91.7% (81-98%) | 7.4% (1-18%) | **2.5% (<2.5%)** |
| CHD25 | 6.1% (3-10%) | 95.8% (81-98%) | 3.2% (1-18%) | **2.7% (<2.5%)** |
| CHD26 | 8.0% (3-10%) | 86.7% (81-98%) | 12.5% (1-18%) | 1.4% (<2.5%) |
| CHD27 | 7.4% (3-10%) | 82.8% (81-98%) | 16.4% (1-18%) | 2.0% (<2.5%) |

^1^ Absolute numbers in cell/µL. Age-matched reference values are shown in brackets [15]. Values below or above the age-matched reference values are shown in **bold**.

^2^ Relative numbers in percentages of T-cell subpopulations within CD3+, CD4+ or CD8+ T-cells. Age-matched reference values are shown in brackets [15]. Values below or above the age-matched reference values are shown in **bold**.

n/a, age-matched reference value not available
